# Water-Preserving and Salt-Resistant Slow-Release Fertilizers of Polyacrylic Acid-Potassium Humate Coated Ammonium Dihydrogen Phosphate

**DOI:** 10.3390/polym13172844

**Published:** 2021-08-24

**Authors:** Hongping Li, Lanwen Yang, Jianxin Cao, Chenchen Nie, Hao Liu, Juan Tian, Wenxing Chen, Pinglan Geng, Guiming Xie

**Affiliations:** 1 School of Chemistry and Chemical Engineering, Guizhou University, Guiyang 550025, China; hongpli547@163.com (H.L.); lanwen958@126.com (L.Y.); jxcao@gzu.edu.cn (J.C.); ncc15139056557@163.com (C.N.); haoliu2019liu@163.com (H.L.); 2 Key Laboratory of Guizhou Province for Green Chemical Industry and Clean Energy Technology, Guizhou University, Guiyang 550025, China; 3 Guizhou Research Institute of Chemical Industry, Guiyang 550002, China; rtr1204@126.com (J.T.); vincene@126.com (W.C.); 4 Guizhou Institute of Products Quality Inspection & Testing, Guiyang 550002, China; Gengpinglan717@163.com

**Keywords:** polyacrylic acid-potassium humate, water retention, salt resistance, ammonium dihydrogen phosphate, slow-release fertilizer

## Abstract

Polyacrylic acid (PAA) has high water absorbency but poor salt resistance. Humic acid (HA) extracted from lignite was introduced into the cross-linked copolymer systems of AA to improve the water absorbency and salt-tolerance. A polyacrylic acid-potassium humate (PAA-KHA) coated ammonium dihydrogen phosphate (ADP) fertilizer with water-preserving, salt-resistant and slow-release properties was prepared. The main properties of HA extracted from lignite oxidized by H_2_O_2_ were studied. Furthermore, the synthesis process, water absorbency of PAA-KHA in deionized water and in NaCl solution, morphologies of PAA-KHA, and the slow-release performance of the fertilizer (ADP@PAA-KHA) were investigated. The results showed PAA-KHA had a layered interpenetrating network, which can provide sufficient storage space for water and nutrients. The salty water absorbency of PAA-KHA increased by about 3 times compared to PAA. Both the PO_4_^3−^ and NH_4_^+^ cumulative release of ADP@PAA-KHA with a coating rate of 10% in deionized water, were less than 20% within 24 h, and were 55.71% and 28.04% after the 15th day, respectively. The weight change of ADP@PAA-KHA before and after absorbing water was about 53 times in deionized water and about 4 times in 1 wt% of NaCl salty water. The results show that ADP@PAA-KHA has excellent properties of water retention, salt resistance and slow-release. This will efficiently improve the utilization of fertilizer and reduce the irrigation water consumption at the same time.

## 1. Introduction

Chemical fertilizers became a useful method to increase grain production to meet the food needs of the rapidly growing global population. However, the direct application of chemical fertilizer causes the loss of fertilizer nutrients due to volatilization of nitrogen in the forms of NH_3_ and nitrous oxides, and leaching in the soil, leading to the rising cost of food production and environmental pollution [1]. Furthermore, water resources become increasingly scarce with global warming, and the dry land without irrigation, such as arid and semi-arid land, accounts for a high proportion of the total arable land [2], Therefore, agricultural development must take the course of sustainable development of water saving, fertilizer reduction and pollution control. Amazingly, the water-preserving and slow-release fertilizer not only makes the release of nutrients match requirements of crops at every stage of growth and development, but also can improve fertilizing, use efficiency to reduce irrigation water consumption and the risk of environmental pollution [3].

Superabsorbent polymers (SAPs) are conducive to use as the coated materials to produce the water-preserving and slow-release fertilizer because the high-water absorbency and the ability to sustain the absorbed water under long time pressure [1]. Furthermore, advantages of SAPs coating materials include improving soil, reducing soil degradation, limiting the evaporation loss, alleviating environmental pollution, lowering crop mortality rates, and enhancing the retention period of plant nutrients in the soil [4]. On the industrial scale of SAPs, acrylic acid (AA) and acrylamide (AM) are the major and common monomers widely employed by copolymerization [5,6,7]. Polyacrylic acid (PAA) and polyacrylamide (PAM) are considered the most widely used water retention agents because of superior water absorbency in water (about 300–500 g·g^−1^) and water retention capacity in synthetic polymers [8]. Nevertheless, the effectiveness of PAA and PAM is hampered by low salt resistance (less than 100 g·g^−1^ in 0.9 wt% of NaCl solution) due to its single type of hydrophilic group [9], low gel strength after water absorption [10], and poor biodegradation [8]. Therefore, of interest are effective approaches to improve their salt-tolerance, mechanical properties, and degradability, such as introduction of kaolin and bentonite to enhance mechanical performance of PAA or PAM polymers [7,11]. The nature polymers can be introduced into the cross-linked copolymer systems of AA or AM [8], such as cellulose [12], natural rubber [13], starch [14,15] and chitosan [16], to improve the degradability, biocompatibility and environmental friendliness. However, SAPs based on natural polymers, such as cellulose, starch and chitosan, are prone to degradation resulting in a short cycle of nutrient release and relatively low slow-release performance [17], and suffer from low water absorption rate, thus have to be used in larger amounts. In addition, most of them are composed of multiple (more than three) substances [18,19,20,21], which complicates the preparation processes and increases their cost.

Humic acid (HA) is an organic carrier and synergist of plant nutrition, which plays a positive role in plant growth [22]. At the same time, HA is a kind of environment-friendly substance with abundant source, low price, safety and non-toxicity [23]. HA is composed of aromatic rings, bridges (including alkylene, ether or ester linkages) and side chains (including alkyl, carboxyl and hydroxyl groups) [24], and it has high chemical reactivity in soil, such as ion exchange, complexation and oxidation-reduction, due to its various active groups and large specific surface area [25], thus it can be introduced into the SAPs to improve water absorbency and salt resistance of the polymer to enhance the water retention capacity and chemical properties of the soil, such as its ability to adsorb soluble salts in the soil, hinder harmful cations, reduce soil salt concentration and so on [26]. Furthermore, Shen et al. [27] showed that bentonite was modified by introducing HA into the interlayer space of bentonite, which exhibited a much higher adsorption capacity to NH_4_^+^-N and NO_3_^−^-N in soil. Previous research in our group also showed that HA has strong adsorption on PO_4_^3−^ and NH_4_^+^ [28]. Therefore, introduction of the HA in the SAPs as coating materials not only can effectively improve the performance of water absorbency and salt resistance, but also can promote the absorption of nutrients by plants.

Lignite is rich in resources [29], cheap in price, and has a high content of HA organic matter. On the basis of the above background and our previous studies [28], in this paper, the HA was extracted from lignite with H_2_O_2_ as an oxygenation agent. Then a coating material with high water absorbability and salt resistance was obtained by the aqueous solution polymerization of acrylic acid and potassium humate. The effects of synthetic conditions on the water absorbency and salty water absorbency of coating materials and the sustained-release performance of coated ammonium dihydrogen phosphate fertilizer were investigated.

## 2. Materials and Methods

### 2.1. Materials

Lignite was obtained from Anshun of Guizhou province, China. Acrylic acid (AA), ammonium persulfate (APS), and soluble starch were purchased from Tianjin Kemiou Chemical Reagent Co., Ltd., Tianjin, China; hydrogen peroxide solution (H_2_O_2_, 30 wt%), potassium hydroxide (KOH), calcium acetate, barium hydroxide (Ba(OH)_2_), sodium chloride (NaCl), sodium hydroxide (NaOH), ammonium dihydrogen phosphate (ADP) were purchased from Chengdu Jinshan Chemical Reagent Co., Ltd., Chengdu, China; N, N′-methylene amide (MBA) was purchased from Shanghai Yuanye Biotechnology Co., Ltd., Shanghai, China; phenolphthalein, sodium pyrophosphate and ammonium ferrous sulfate were purchased from Tianjin Zhiyuan Chemical Reagent Co., Ltd., Tianjin, China; potassium dichromate (premium pure) was from Beijing Balinwei Technology Co., Ltd., Beijing, China; 1,10-Phenanthroline monohydrate was purchased from Shanghai Ika Biotechnology Co., Ltd., Shanghai, China; Sulfuric acid (H_2_SO_4_, 95–98 wt%) and hydrochloric acid (HCl, 36–38 wt%) were purchased from Chongqing Chuandong Chemical (Group) Co., Ltd., Chongqing, China. All the above chemicals were of analytical grade unless otherwise stated. Deionized water was used in all the experiments.

### 2.2. Methods

#### 2.2.1. Extraction of HA from Lignite by H_2_O_2_ Oxygenation

Lignite was dried and ground to a particle size of less than 0.2 mm, and then 10 g were placed in a 250 mL of beaker; meanwhile, 10 mL of H_2_O_2_ solution was added, and then stirred at 200 rpm for 24 h in the dark at room temperature. After that, oxygenated lignite was obtained by filtering, and drying at 50 °C.

An amount of 5 g of oxygenated lignite was added in 50 mL 2 wt% of KOH solution and then reacted at 400 rpm and 70 °C for 30 min in a three-neck flask. Then the supernatant of the mixture was divided into two parts after separating by the centrifugation. One of the supernatants was blown dry at 60 °C to obtain potassium humate (KHA). The other supernatant had 0.2 mol·L^−1^ of HCl solution added to adjust the pH to 1.5 and stood for 24 h. Thereafter, the sediment obtained by filtration was washed with deionized water until neutral and was dried at 60 °C to acquire HA.

#### 2.2.2. Preparation of PAA-KHA

The typical polymerization process was as follows: 5 mL of deionized water and 2 g of AA monomer (neutralized to 50% degree of neutralization by 0.02 g·mL^−1^ of KOH solution beforehand) were added to a three-neck flask, equipped with a thermometer, a nitrogen line and a reflux condenser. Then 6 wt% of KHA, 2.1 wt% of APS and 0.2 wt% of MBA (based on the AA monomer, respectively) were added under stirring of 500 rpm. Nitrogen was continuously filled in to remove the air, and then the mixture in the flask was kept in a 70 °C water bath for 4.5 h. The reaction was terminated by cooling to room temperature, and a polymer solution with a certain fluidity was obtained, which was directly used as a coating material to prepare slow-release fertilizer. Additionally, the polymer solution was washed, precipitated, and separated by ethanol, then it was dried in an oven at 60 °C, which was used for analysis and characterization.

PAA material was prepared under the same conditions as PAA-KHA except that it did not add the 6 wt% of KHA.

#### 2.2.3. Preparation of ADP@PAA-KHA

Previous research showed that oxygenated lignite has strong adsorption on PO_4_^3−^ and NH_4_^+^ [28], which is of benefit to the absorption of crops. Therefore, soluble starch was selected as the binder and granulated with the oxygenated lignite and ADP to prepare fertilizer cores. The specific preparation process was as follows: 1000 g mixture of oxygenated lignite, ADP and soluble starch with the mass ratio of 4:2:2 was granulated in a rotary granulator (BYC−300, Shanghai Tianhe Machinery Co. Ltd., Shanghai, China) at 30 rpm for 30 min under the condition of intermittent spraying a small amount of water, and then the fertilizer cores were obtained by air-drying, and they were screened. Then 500 g of fertilizer cores were placed again in the rotary granulator and slowly poured into 50 g of PAA-KHA polymer solution at the rotation speed of 35 rpm and kept for 30 min at 60 °C to obtain the ADP@PAA-KHA fertilizers. The coated rate (w%) was calculated according to the Formula (1).

(1) $$w\%=\frac{m_{2}-m_{1}}{m_{1}}\times 100\%$$

where m_1_ (g) and m_2_ (g) was the weight of fertilizer cores and ADP@PAA-KHA, respectively.

### 2.3. Characterization

#### 2.3.1. Determination the Properties of HA

Determination of HA ad (%) in oxygenated lignite was done according to ISO 5073: 1999. The content of the carboxyl groups and total acid groups were determined by calcium acetate titration and barium hydroxide titration respectively, and the content of phenolic hydroxyl groups were the content of the total acid groups minus carboxyl groups [30]. All the titration experiments were conducted over three parallel experiments, and the final experimental results were the average value of the three experiments.

#### 2.3.2. Determination of Water Absorbency of PAA-KHA in Deionized Water and Salty Water

PAA-KHA with mass of M_1_ was accurately weighed and put into a beaker with 1000 mL of deionized water (or 1 wt% of NaCl salty water solution) and underwent full swelling at 25 °C for 24 h. The swollen material was filtered out with a sieve until there was no water dripping and it reached a weight of M_2_. Then the water absorbency of PAA-KHA in deionized and salty water was calculated according to Formula (2). The water absorbency of PAA in deionized water and salty water was tested by the same method as the PAA-KHA. All the experiments were conducted across three parallel experiments, and the final experimental results were the average value of the three experiments.

(2) $$H\left(g\cdot g^{-1}\right)=\left(M_{2}-M_{1}\right)/M_{1}$$

where H (g·g^−1^) was the water absorbency of PAA-KHA in deionized or salty water, M_1_ (g) was the mass of PAA-KHA, and M_2_ (g) was the mass of PAA-KHA after fully swelling in deionized or salty water, respectively.

To analyze the repeated water absorbency of PAA-KHA, the test procedures were as follows: the PAA-KHA was fully swelled in deionized water and its water absorbency was calculated as above. Then the PAA-KHA was dried to a constant weight at 30 °C and put back into the deionized water to fully swell to test the water absorbency again. The procedures were repeated five times. The repeated water absorbency of PAA was tested by the same method as that of the PAA-KHA. A certain amount of PAA-KHA (or PAA) was weighed at 25 °C, placed in water until fully swelled, and then transferred into an incubator at 35 °C. The mass of PAA-KHA (or PAA) was weighed at specific intervals and its water loss was calculated to determine the water retention capacity of PAA-KHA (or PAA). All experiments were conducted in parallel, and the final results were averaged.

#### 2.3.3. Determination of Slow-Release Performance of the ADP@PAA-KHA Fertilizer

The static water immersion method was selected to determine the cumulative release rate of each nutrient element of ADP@PAA-KHA. A certain amount of ADP@PAA-KHA was placed in 500 mL of boiled deionized water in an incubator with a constant temperature of 25 °C. Liquid samples, weighed out at 10 mL at regular intervals, were used to determine the content of nutrient elements, and at the same time another 10 mL of boiled deionized water was replenished to the system. The concentrations of NH_4_^+^ and PO_4_^3−^ were measured by American ion chromatograph (ICS−900, Dionex Company, Sunnyvale, CA, USA), and the cumulative release rate of each nutrient (W) was calculated according to the following Formula (3) [31].

(3) $$W\left(\%\right)=\frac{V_{E}\sum_{1}^{n-1}C_{i}+V_{0}C_{n}}{m_{x}}\times 100\%$$

where W (%) was the cumulative release rate of each nutrient, V_E_ (L) and V_0_ (L) were the volume of sample solution and solution in container, respectively, C_i_ (mg·L^−1^) was the concentration of nutrient in the sample solution, C_n_ (mg·L^−1^) was the concentration of the solution in the container, n and i were the number of samples, respectively and m_x_ (mg) was the nutrient mass in the sample.

An FTIR spectrometer (Nicolet IS50, Thermo Fisher Scientific Company, Waltham, MA, USA) was used to analyze the surface functional groups of lignite, oxygenated lignite by H_2_O_2_, HA extracted from lignite and PAA-KHA [24,32].

Morphologies of PAA-KHA before and after water-absorption were observed using a scanning electron microscope (S−3400N, Hitachi, Japan). The samples were coated with a layer of gold before the microscopic examination. Energy dispersive spectroscopy (EDS, EDAX Octane Prime, Mahwah, NJ, USA) was used to analyze the element composition of the designated region of PAA-KHA. The swollen samples were freeze-dried in a freeze dryer (NAI-T1−5, NAI Precision Instrument Co., Ltd., Shanghai, China) at −50 °C for 48 h before the test.

## 3. Results and Discussion

### 3.1. Extraction of HA from Lignite

**Figure 1:**
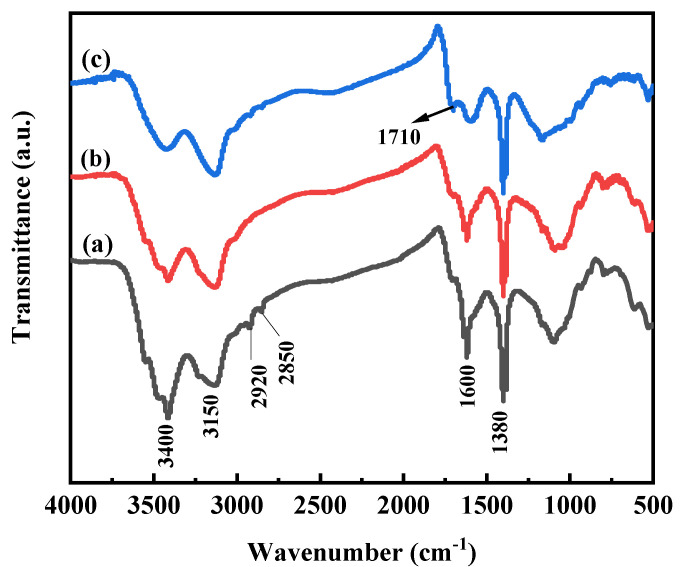
The FTIR spectra of lignite (a), oxygenated lignite (b) and HA(c).

As shown in Table 1, after lignite oxygenation by H_2_O_2_, the total acid groups’ content of lignite increased from 4.63 to 10.63 mmol·g^−1^, while the carboxyl groups content increased from 2.35 to 3.20 mmol·g^−1^. In addition, the phenolic hydroxyl groups’ content increased from 2.28 to 7.60 mmol·g^−1^ and the HA content increased from 18.78% to 35.13%, a significant increase of 16.35%. The results showed that H_2_O_2_ was as an outstanding oxidant of lignite, which significantly increased the HA production from lignite. The chemical structure differences of lignite, oxygenated lignite and HA are characterized by FTIR in Figure 1; the broadband at around 3400 cm^−1^ belongs to the stretching vibration of the -OH groups, and the broadband at around 3150 cm^−1^ belongs to the N-H or =C-H of the aromatic ring. The peaks occurring at about 2940–2920 and 2850 cm^−1^ are attributed to the asymmetric and symmetric aliphatic C-H stretching of CH_2_ groups. An intense peak at 1725–1710 cm^−1^ is attributed to C=O stretching of COOH. The peaks at 1600 cm^−1^ corresponded to the stretching vibration of C=C in the aromatic nucleus. A peak at around 1380 cm^−1^ can be attributed to C-H deformation of CH_2_ and CH_3_ groups and/or to antisymmetric stretching of COO- groups [24,32,33,34] These results showed that a variety of functional groups existed on HA and they were well-preserved after being oxidized by H_2_O_2_, which indicates that H_2_O_2_ is a suitable oxygenation agent for lignite.

### 3.2. Effect of Synthesis Conditions on the Water Absorbency of PAA-KHA

**Figure 2:**
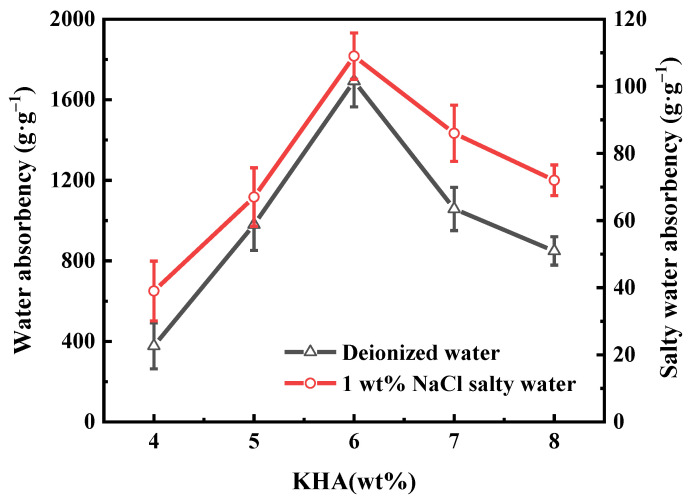
The effect of KHA content on the water absorbency of PAA-KHA in deionized water and in 1 wt% of NaCl salty water (2 g of AA, 50% degree of neutralization, 2.1 wt% of APS and 0.2 wt% of MBA).

The impacts of KHA content on the water absorbency of PAA-KHA in deionized water and in 1 wt% of NaCl solution are shown in Figure 2. When the KHA content was lower than 6 wt%, it was obvious that water absorbency increased with the increase of KHA content and the peak occurred at 6 wt%. The main reason could be that the number and types of hydrophilic groups on the polymer chain were richer on KHA. The synergistic effect between the groups formed hydrogen bonds with water molecules, which significantly improved the hydrophilicity of the PAA-KHA material and promoted the water absorption behavior. Below the concentration of 6 wt%, the active sites in the system were insufficient and the excess AA could be caused by self-polymerization, and the resulting polymer structure was too dense to form the three-dimensional network structure, which was not conducive to water absorption [35]. However, when the amount of KHA was too high, the water absorption of PAA-KHA was diminished, possibly due to excess KHA which could not interlace into the graft copolymer, resulting in part of the three-dimensional network structure of polymer occupied or blocked by the macromolecular KHA, reduced permeability between the inside and outside of the polymer network, and hindered water molecule infiltration [33].

**Figure 3:**
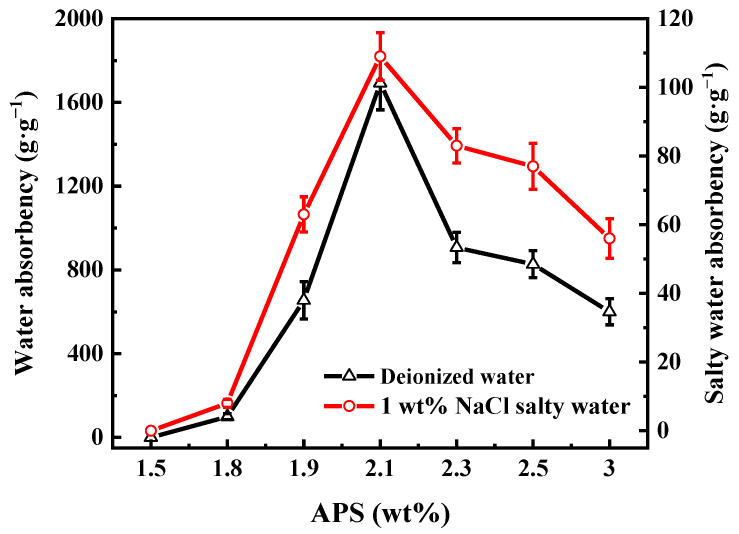
The effect of APS content on the water absorbency of PAA-KHA in deionized water and in 1 wt% of NaCl salty water (2 g of AA, 50% degree of neutralization, 6 wt% of KHA and 0.2 wt% of MBA).

The impacts of APS content on the water absorbency of PAA-KHA in deionized water and in 1 wt% of NaCl solution are shown in Figure 3. Both water absorbencies of PAA-KHA reached a peak at 2.1 wt% of APS. The initiator affects the number of active sites in the reaction system [35]. When the content of APS was lower than 1.5 wt%, there were fewer active sites produced. As a result, the polymerization reaction could not be initiated or the degree of polymerization reaction was much limited. When APS was 1.8 wt%, although the polymerization reaction could be carried out, there were many remaining unreacted monomers in the system. Moreover, the polymer formed had a low molecular weight and a low degree of network crosslinking, leading to its poor absorbing ability of water or NaCl solution. Then, the water absorbency increases with increasing APS content from 1.8 to 2.1 wt%, the APS provided more active sites, accelerated the grafting reaction between KHA and AA, improved the three-dimensional network structure, and enhanced water absorption [35]. However, when the content of APS was higher than 2.1 wt%, the water absorbency decreased with increase of APS concentration. Because in free-radical chain polymerization using a chemical initiator, the number-average degree of polymerization is inversely proportional to the square root of the concentration of the initiator, the molecular weight of grafting on the polymer backbone would decrease, and the relative amount of polymer chain, which does not contribute to the absorption capacity, ends the increase [36]. Moreover, the excess APS initiator accelerated AA self-polymerization, as indicated by shorter chain length in the product, and the resulting polymer was too dense to form the three-dimensional network structure, which was not conducive to water absorption [35].

**Figure 4:**
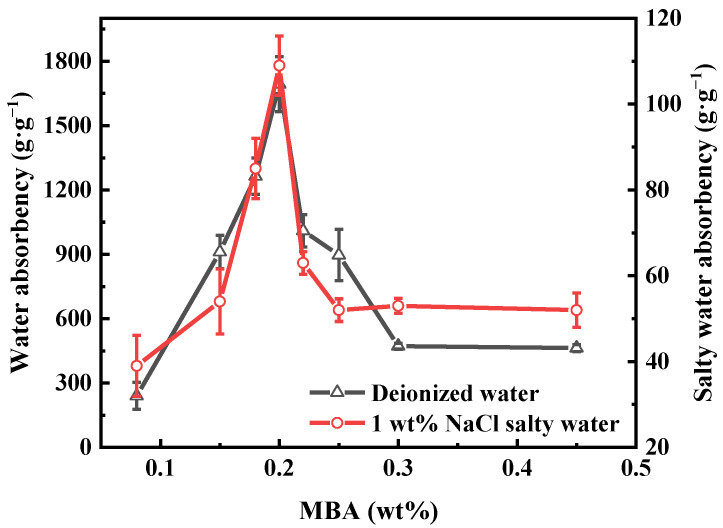
The effect of MBA content on the water absorbency of PAA-KHA in deionized water and in 1 wt% of NaCl salty water (2 g of AA, 50% degree of neutralization, 6 wt% of KHA and 2.1 wt% of APS).

Different wt% of MBA ranging from 0.1 to 0.45 wt% were used to determine the impacts of the crosslinking agent on PAA-KHA. As shown in Figure 4, with the increase of MBA content, both water absorbency of PAA-KHA in deionized water and in 1 wt% of NaCl solution increased first and then decreased. There was a maximum water absorbency when the MBA content was 0.2 wt%, which was 1693 g·g^−1^ in deionized water and 109 g·g^−1^ in NaCl solution, respectively. PAA-KHA failed to form a rich crosslinking network structure or strong network structure when the MBA content was relatively low. The low content of MBA made the polymer more water-soluble, and eventually led to a lower absorption ability. Additionally, the decrease of water absorbency when the MBA content exceeded 0.2 wt% could be attributed to the abundant crosslinking points in the structure. The high crosslinking density caused the shrinkage of micropores in the network structure, which limited the relaxation in the water.

**Figure 5:**
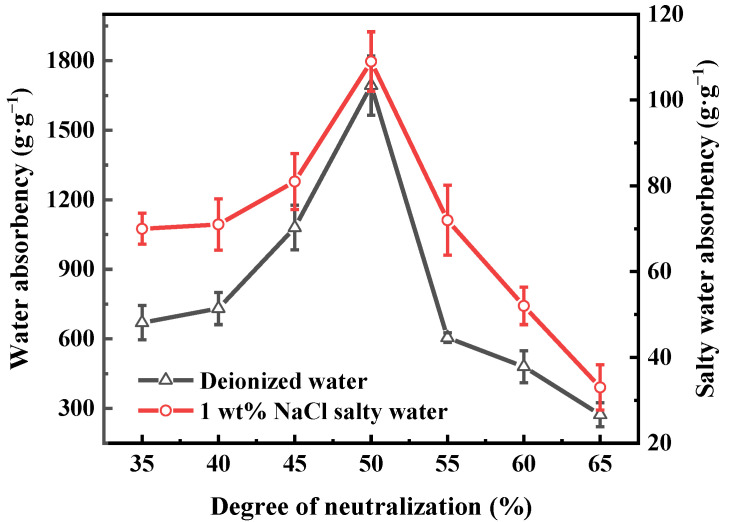
The effect of degree of neutralization on the water absorbency of PAA-KHA in deionized water and in 1 wt% of NaCl salty water (2 g of AA, 6 wt% of KHA, 2.1 wt% of APS and 0.2 wt% of MBA).

The direct addition of AA makes explosive polymerization easier due to the high activity of the AA monomer. Therefore, KOH was used to neutralize with –COOH in the AA part. The generated potassium acrylate had a slower polymerization reaction rate and let the reaction proceed more smoothly. Figure 5 shows that the maximum water absorbency of PAA-KHA was found at 50% degree of neutralization. When the neutralization degree was lower than 50 wt%, the system was more acidic, the reaction was faster, and the crosslinking density of the polymer was higher, which resulted in lower water absorbency. Conversely, the excessive K^+^ ionization in the system made the concentration of –COO^−^ too high when the degree of neutralization was greater than 50 wt%. Therefore, the free motion of polymer chains were limited by mutual repulsion, which limited the expansion of the network microporous structure.

### 3.3. Properties of PAA-KHA

**Figure 6:**
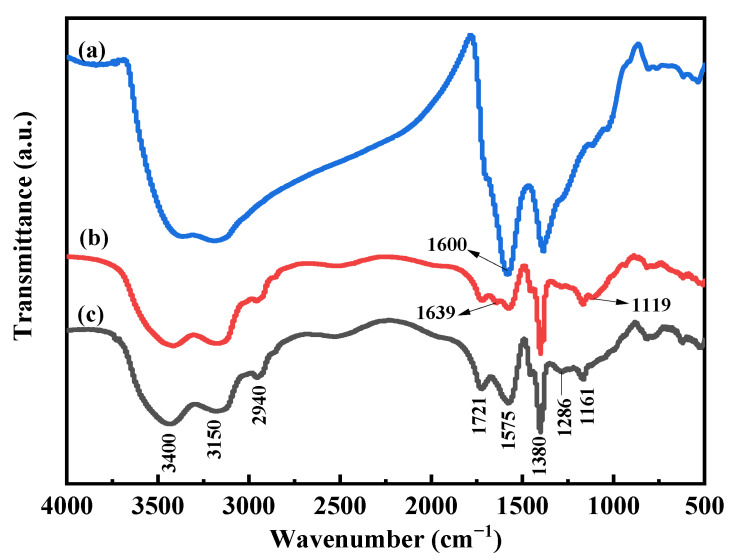
The FTIR spectra of KHA (a), PAA (b) and PAA-KHA (c).

The FTIR spectra of PAA-KHA are presented in Figure 6; the broad absorption band of PAA-KHA at 3400, 3150 and at 2940 cm^−1^ appeared due to the stretching vibration of –OH, the N-H or =C-H of the aromatic ring and stretching vibration of aliphatic C-H, respectively. The peak occurring at about 2940 cm^−1^ is attributed to the asymmetric and symmetric aliphatic C-H stretching of CH_2_ groups. The peaks of 1721 cm^−1^ indicated the stretching vibration of C=O originated from –COOH. The adsorption band at 1575 cm^−1^ in the PAA-KHA spectrum was attributed to the stretching vibration of C=C from a large number of extended aromatic rings in potassium humate. Additionally, the sample also exhibited several characteristic signals, such as the C-H deformation of CH_2_ and CH_3_ groups and/or to antisymmetric stretching of COO- groups at 1380 cm^−1^, the vibrating absorption of the alkyl aryl at 1286 cm^−1^, and the vibrating absorption of acrylate –COO- at 1161 cm^−1^ [32]. Due to the interaction between the groups of KHA and PAA, some absorption peaks of PAA-KHA can be observed to become stronger (such as at the peak of 2940, 1721, 1575 and 1286 cm^−1^) or disappear (such as the stretching vibration of aliphatic C-H of the C=C which originated from the PAA at the peak of 1639 cm^−1^) compared to the PAA indicating that the reaction has taken place. The obtained PAA-KHA polymer also was verified by SEM and EDS in the later section.

**Figure 7:**
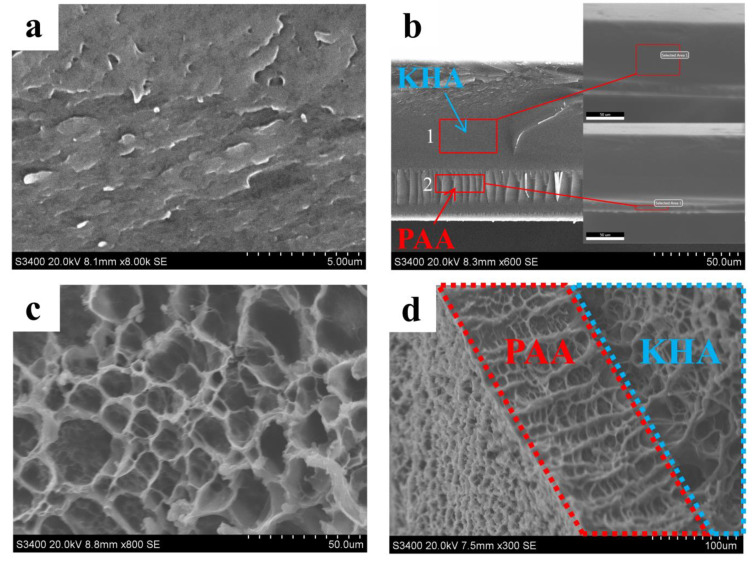
SEM images of PAA-KHA: (**a**) surface before water absorption, (**b**) section before water absorption, (**c**) surface after water absorption, and (**d**) section after water absorption.

SEM revealed the surface morphologies of PAA-KHA in Figure 7. The surface of PAA-KHA before water absorption was similar to the irregular fish scales in Figure 7a. The PAA-KHA section morphology presented a layered state, in which the upper layer was relatively smooth, and the lower layer presented an orderly vertical pore structure, which provided channels for the water absorption in Figure 7b. EDS analysis of the PAA-KHA material section showed that there were differences in the element composition of the area where each layer was located, as shown in Table 2. It can be inferred from the element content that the first layer was mainly KHA, while the second layer was mainly PAA. The layered structure of the PAA-KHA material was beneficial to the water absorption and water retention of material, while the vertical pores provided channels for water absorption of material. In addition, the interpenetrating network structure in the KHA layer acted as a micro reservoir, which provided a water storage container for water storage after the material absorbs water. The SEM of freeze-dried PAA-KHA after absorption water and saturation is shown in Figure 7c,d, the PAA-KHA was freeze-dried after water absorption saturation and the corresponding PAA layer and KHA layer pore channels were opened. Due to the rich pore structure, the PAA-KHA as the coating material of fertilizer can maintain a good water absorption and retention effect.

**Figure 8:**
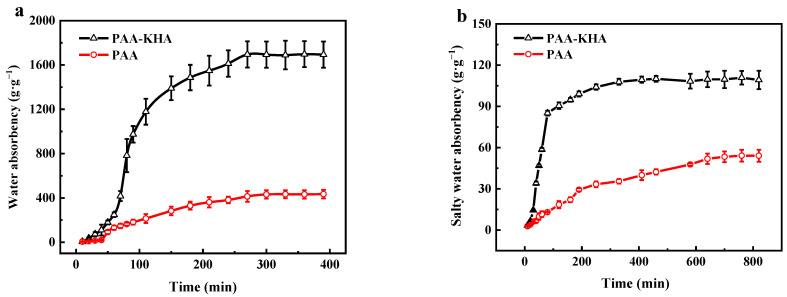
Water absorbency of PAA (2 g of AA, 2.1 wt% of APS and 0.2 wt% of MBA) and PAA-KHA (2 g of AA, 6 wt% of KHA, 2.1 wt% of APS and 0.2 wt% of MBA) in deionized water (**a**) and in 1 wt% of NaCl salty water (**b**), respectively.

Figure 8 shows the curve of the water absorbency of PAA and PAA-KHA in deionized water and in 1 wt% of NaCl solution, respectively. The water absorbency of PAA-KHA increased with time increment in an “S” shape and the water absorbency reached the saturation state in about 230 min and 200 min, respectively. The water absorption process consists of three stages: In the first stage, the water absorbency is slow because the water molecules entered the porous interpenetrating network structure and made full contact with it when PAA-KHA was in contact with water, so that the interaction between ions was small at the time. In the second stage, the water absorbency improved due to the strong hydration between hydrophilic groups and water molecules when PAA-KHA began to absorb water, and the ion concentration of the solution in the porous interpenetrating network structure was very high, resulting in a large osmotic pressure difference between the inside and outside of the solution. In the third stage, the water absorbency slowed down again as the ion concentration of the solution decreased and the osmotic pressure difference between the inside and outside decreased after a large amount of water molecules entering the PAA-KHA structure. Finally, the water absorbency slows down to saturation because of the binding effect of the polymer chain. In addition, the water absorbency of PAA-KHA was much higher than that of PAA whether it was in deionized water or salt solution, while the time required to reach the absorption saturation of PAA-KHA was shorter than that of PAA, which proved that PAA-KHA had better and faster water absorption capacity. The PAA-KHA material coated fertilizer may reduce the irrigation time and frequency in agriculture.

**Figure 9:**
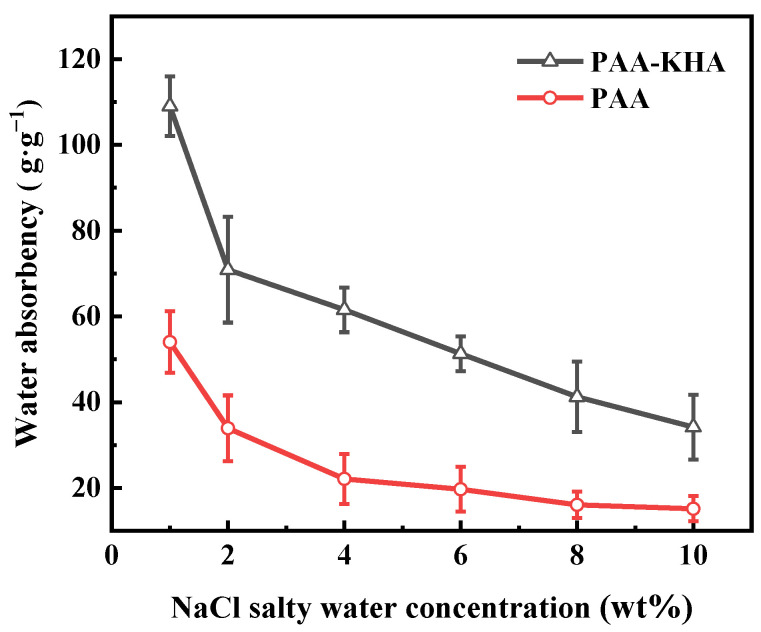
The effect of NaCl salty water concentration on water absorbency of PAA and PAA-KHA.

As seen in Figure 9, the water absorbency of PAA and PAA-KHA decreased with the increase of NaCl solution concentration from 1 to 10 wt%, respectively. The reason was that after the PAA and PAA-KHA absorbed salty water they were in a gel state, the ion concentration in the solution increased, so that the shielding effect between ions increased and the osmotic pressure difference between inside and outside the pore structure decreased, which limited the expansion of their network structure and led to the decrease of water absorbency. In the same concentration of NaCl solution, the water absorbency of PAA-KHA was about 3 times higher than that of PAA. For example, the water absorbency of PAA-KHA was 61.3 g·g^−1^ when the concentration of NaCl salty water was 4 wt%, while PAA was only 22.5 g·g^−1^. These results demonstrated that the addition of HA significantly improved the salt resistance of the coating material regardless of the concentration of salt solution.

**Figure 10:**
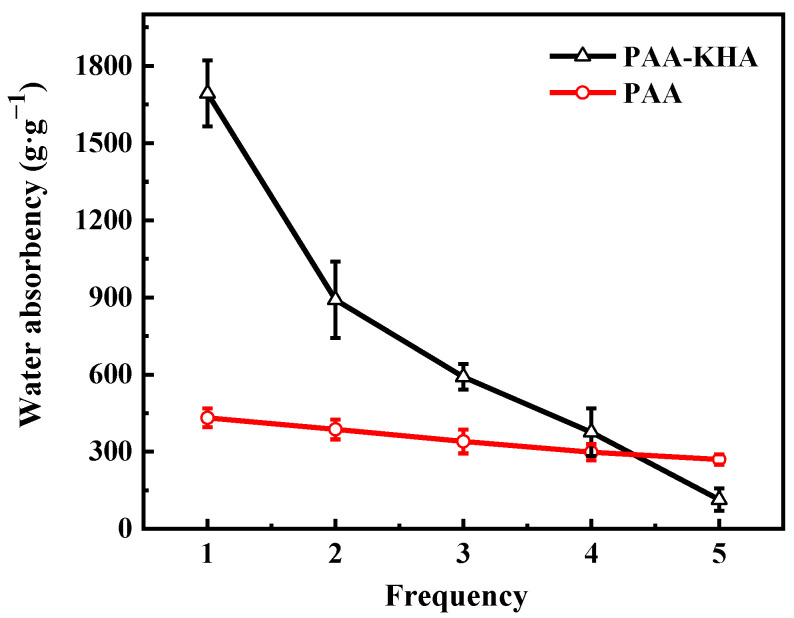
The repeated water absorbency of PAA and PAA-KHA.

**Figure 11:**
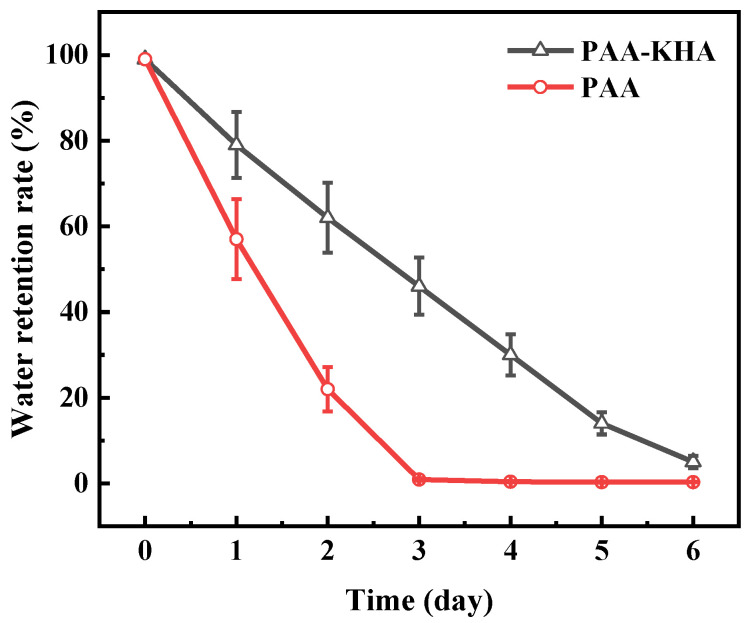
Water retention of PAA and PAA-KHA.

The repeated water absorbency of the PAA and PAA-KHA are shown in Figure 10, respectively. The water absorbency of PAA and PAA-KHA decreased with the increase of water absorption frequency. Compared with PAA, the decreased rate of PAA-KHA was relatively faster. The water absorbency of PAA-KHA after the fifth repetition was 114 g·g^−1^, only 6.73% of the water absorbency of the first time, while that of PAA was 62.5% of the water absorbency of the first time. This phenomenon may be caused by the partial destruction of PAA-KHA structure during the repeated water absorption process. The PAA-KHA weight is only 28% of the original sample after the fifth repetition of water drying, which proved that part of the polymer may be partially dissolved in water, and indirectly indicates that PAA-KHA, as a fertilizer coating material, has degradability in the soil environment. Figure 11 shows that both PAA and PAA-KHA water retention rates presented a downward trend with time, and the steepness of PAA-KHA was slower than PAA. PAA lost almost all of its water by the third day, while PAA-KHA still had 5% of water content after six days. It proved that the introduction of KHA not only increased the hydrophilic group of the material, but also introduced the internal multi-pore interpenetrating network structure of the PAA-KHA, shown in Figure 7. This structure enabled PAA-KHA to have a larger contact area with water molecules, thus enhancing the hydrophilicity and water retention performance of PAA-KHA.

### 3.4. Properties of ADP@PAA-KHA Fertilizer

**Figure 12:**
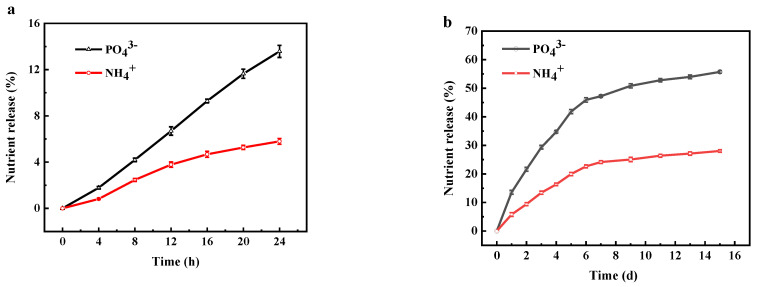
The cumulative release rate of ADP@PAA-KHA at different times, (**a**) 24 h, and (**b**) 15 days.

ADP@PAA-KHA sample with coating rate of 10% was selected, and its slow-release performance for 24 h and 15 days are shown in Figure 12, respectively.

**Figure 13:**
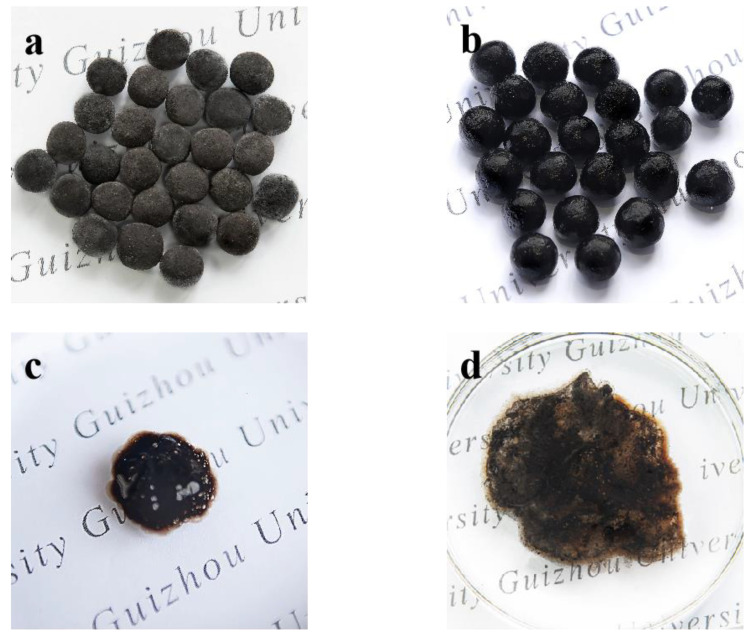
Electronic digital photograph of fertilizer: (**a**) fertilizer core, (**b**) ADP@PAA-KHA, (**c**) ADP@PAA-KHA absorbs water for 20 min, and (**d**) ADP@PAA-KHA absorbs water for 24 h.

In Figure 12, the release rate of PO_4_^3−^ was much higher than that of NH_4_^+^ in static water. The cumulative release rate of nutrients in the ADP@PAA-KHA after 24 h was lower than 20%. The release of nutrients was very slow in the initial 4 h, because the pore structure network of PAA-KHA was not sufficiently open in the early stage; it can be seen from the morphology of the ADP@PAA-KHA absorbed water for 20 min in Figure 13c, while the interpenetrating network structure of PAA-KHA has not yet opened by the intake of water. The nutrients were slowly released and dissolved in the aqueous solution when the network structure was filled with water after 4 h. According to Figure 12b, the releasing rates of PO_4_^3−^ and NH_4_^+^ were relatively fast in the first 6 days. This was because the three-dimensional interpenetrating network structure was fully opened after the PAA-KHA material absorbed adequate water and the concentration gradient of internal and external was large, which finally accelerated the nutrients release rate. As shown in Figure 7d and Figure 13d, the pore structure of PAA-KHA was completely opened after water absorption and saturation. The cumulative release of PO_4_^3−^ and NH_4_^+^ were 45.92% and 22.65% on first 6 days, respectively. The cumulative release rates of PO_4_^3−^ and NH_4_^+^ were 55.71% and 28.04% after the 15th day, respectively. It can be seen that the nutrients release rate slows down obviously, which indicated that the fertilizer had good slow-release characteristics.

The morphologies and water absorption performance of ADP@PAA-KHA are shown in Figure 13. The surface of the coated fertilizer (Figure 13b) became smoother after being embedded by PAA-KHA than the surface before coating (Figure 13a). After the ADP@PAA-KHA absorbed water for 20 min, the interpenetrating network structure of PAA-KHA had not yet opened by swelling in process of contacting with water, and the surface with sufficient contact with water showed a burr shape due to water absorption (Figure 13c). However, the water completely entered the interpenetrating network structure, which formed a miniature reservoir (Figure 13d) after 24 h. The weight change of ADP@PAA-KHA with a 10% coating rate before and after absorbing water in deionized water was about 53 times, and it was about 4 times in 1 wt% of NaCl salty water. The results showed that the ADP@PAA-KHA had prominent performance on water retention and salt tolerance.

## 4. Conclusions

The study selected lignite as the source, H_2_O_2_ as the oxidant to oxygenize lignite and then HA was extracted from lignite. Then the HA and AA monomers were used to prepare a coating material of PAA-KHA by aqueous solution polymerization. The optimum preparation conditions were 50% degree of neutralization of 2 g of AA with a composition of 6 wt% of KHA, 2.1 wt% of APS and 0.2 wt% of MBA. PAA-KHA had a layered interpenetrating network, which can provide sufficient storage space for water and nutrients. The water absorbency of PAA-KHA was as high as 1693 g·g^−1^ and 109 g·g^−1^ in deionized water and 1 wt% of NaCl salty water solution. The salt resistance of PAA-KHA increased by about 3 times that of PAA. The water absorbency remained at 114 g·g^−1^ after 5 re-absorptions. There was still 5% of water content after 6 days exposure in the air at 35 °C. Both the PO_4_^3−^ and NH_4_^+^ cumulative release of ADP@PAA-KHA with coating rate of 10% in deionized water, were less than 20% within 24 h. The cumulative release rates of PO_4_^3−^ and NH_4_^+^ were 55.71% and 28.04% within 15 days, respectively. The weight change of ADP@PAA-KHA before and after absorbing water was about 53 times in deionized water and about 4 times in 1 wt% of NaCl salty solution. The ADP@PAA-KHA has excellent properties of water retention, salt-resistance and slow-release, and it could efficiently improve the utilization of fertilizer and reduce the irrigation water consumption at the same time.

## Figures and Tables

**Table 1:** The characteristics of lignite and oxygenated lignite.

Content	Lignite	Oxygenated Lignite
Total acid groups (mmol·g^−1^)	4.63	10.63
Carboxyl (mmol·g^−1^)	2.35	3.20
Phenolic hydroxyl (mmol·g^−1^)	2.28	7.60
HA ad (%)	18.78	35.13

**Table 2:** Element composition of different areas of PAA-KHA.

Regions	Element Mass Percent (wt%)			Atom Percent (at%)		
	C	O	K	C	O	K
The first layer	31.91	40.73	27.35	45.01	43.13	11.85
The second layer	39.57	39.60	20.83	52.28	39.27	8.45

## Data Availability

The data used to support the findings of this study are included within the article.

## Nomenclature

PAA	Polyacrylic acid
HA	Humic acid
KHA	Potassium humate
PAA-KHA	Polyacrylic acid-potassium humate
ADP	Ammonium dihydrogen phosphate
ADP@PAA-KHA	Polyacrylic acid-potassium humate coated ammonium dihydrogen phosphate fertilizer
AA	Acrylic acid
APS	Ammonium persulfate
MBA	N, N′-methylene amide
w	coated rate (%)
m_1_	the weight of fertilizer cores (g)
m_2_	the weight of ADP@PAA-KHA (g)
H	water absorbency (g·g^−1^)
M_1_	the mass of PAA-KHA (g)
M_2_	the mass of PAA-KHA after fully swelling in deionized or salty water (g)
W	the cumulative release rate of each nutrient (%)
V_E_	the volume of sample solution (L)
V_0_	the volume of solution in the container (L)
C_i_	the concentration of nutrient in the sample solution (mg·L^−1^)
C_n_	the concentration of the solution in the container (mg·L^−1^)
n and i	the number of samples
m_x_	the nutrient mass in the sample (mg)
